# What Role Do Perfectionism and Cognitive Pre‐Sleep Arousal Play in the Link Between Stress and Sleep? A Daily Diary Study in University Students

**DOI:** 10.1002/smi.70136

**Published:** 2026-02-05

**Authors:** Alexander Haussmann, Nina Schilling, Marie Alfter, Jannis Yahja, Alica Mertens, Laura I. Schmidt

**Affiliations:** ^1^ Department of Gender Studies and Health Psychology Institute of Psychology Heidelberg University Heidelberg Germany; ^2^ Section of Health Care Research and Rehabilitation Research Institute of Medical Biometry and Statistics Faculty of Medicine Medical Center University of Freiburg Freiburg Germany; ^3^ Section of Cognitive Psychology and Cognitive Aging School of Social Science University of Mannheim Mannheim Germany

**Keywords:** actigraphy, arousal, daily diary, ecological momentary assessment, perfectionism, sleep, stress, students

## Abstract

Insufficient sleep is common among university students and impairs health and academic functioning. While multidimensional perfectionism (perfectionistic concerns and strivings) and daily stress are potential contributors, yet their interplay and underlying cognitive mechanisms remain unclear. Cognitive pre‐sleep arousal may mediate links between stress, personality traits, and sleep. In a 14‐day micro‐longitudinal study, 88 German university students (*M* = 22.47 years, SD = 3.48) wore fitness trackers and completed daily diaries assessing objective sleep duration, subjective sleep quality, subjective sleep onset latency (SOL), daily stress, and cognitive pre‐sleep arousal. Trait perfectionism and covariates (emotional distress, Big Five traits, and sex) were measured via questionnaires. Multilevel modelling and structural equation modelling were used. Neither perfectionistic concerns nor strivings predicted any sleep parameters. However, daily stress was associated with shorter sleep duration (*b* = −0.21, *p* = 0.033), lower sleep quality (*b* = −0.09, *p* = 0.006), longer SOL (root transformed: *b* = 0.01, *p* = 0.046), and higher cognitive arousal (*b* = 0.06, *p* < 0.01). No interaction effects between perfectionism and stress were found. Within‐person mediation showed that on days with elevated stress, increased cognitive pre‐sleep arousal partially explained shorter sleep (indirect effect = −0.16), lower sleep quality (indirect effect = −0.08), and longer SOL (indirect effect = 0.01; all *p* < 0.001). Exploratory analyses indicated that emotional distress, unlike perfectionism, predicted longer SOL via heightened cognitive pre‐sleep arousal (indirect effect = 0.09, *p* = 0.007). Given the suboptimal model fit in the mediation models, all indirect effects should be interpreted with caution. Daily stress robustly impairs sleep and elevates cognitive pre‐sleep arousal, which partially mediates its negative effects on sleep variables. Multidimensional perfectionism was not associated with sleep, nor did it moderate the stress‐sleep link. Targeting cognitive pre‐sleep arousal may be a promising mechanism to improve sleep in students experiencing elevated stress.

## 1 Introduction

Insufficient sleep is detrimental to human physical and mental health. Consequences of poor sleep range from short‐term consequences (e.g., increased stress reactivity, somatic problems, reduced quality of life) to long‐term risks, such as cardiovascular diseases and diabetes (Johnson et al. 2021; Medic et al. 2017). On the other hand, improving sleep can lead to substantial improvements in mental health, including symptoms of depression and anxiety (Scott et al. 2021). However, young people often report insufficient sleep (Kolip et al. 2022), with university students appearing to be particularly affected by poor sleep quality. A study of over 7626 U.S. students found that 27% rated their sleep quality as poor, and 62% met the cutoff criteria for poor sleep quality on the Pittsburgh Sleep Quality Index (PSQI; Becker et al. 2018). Similar findings were observed in a sample of 1684 German students, where 49% of participants reported impaired sleep quality based on the PSQI (Schmickler et al. 2023). Beyond health, sleep also plays a vital role in memory consolidation, cognitive functioning, and academic performance (Alhola and Polo‐Kantola 2007; Okano et al. 2019; Wardle‐Pinkston et al. 2019). Students may therefore be particularly vulnerable to the negative consequences of sleep problems. In order to develop effective interventions in the future, it is important to identify factors associated with insufficient sleep among students. Given the high prevalence of stress in university life and the importance of cognitive processes for sleep, we focus on perfectionism as a potential vulnerability factor that may intensify stress‐related cognitive activity and thereby impair sleep quality.

Perfectionism is one of the common personality constructs investigated in relation to poor sleep (Stricker et al. 2022). The Perfectionism Cognition Theory (PCT; Flett et al. 2015) offers a useful framework for understanding how a perfectionistic mindset shapes cognitive processes and behaviours in ways that may also contribute to poor sleep. According to the PCT, perfectionism reflects a cognitive‐dispositional vulnerability characterised by excessively high personal standards alongside heightened self‐focused and evaluative thinking. This combination fosters perseverative cognitive activity, leading to recurrent worry, rumination, and other forms of intrusive, self‐critical thought (Xie et al. 2019). Notably, these cognitive tendencies closely parallel the dysfunctional processes emphasised in cognitive models of insomnia, which identify excessive worry, preoccupation with sleep, and difficulties in down‐regulating mental activity at bedtime as core mechanisms underlying poor sleep (Espie 2007; Harvey 2002; Tang et al. 2023). Given this conceptual overlap with cognitive models of insomnia, it is crucial to identify which aspects of perfectionism are most likely to elicit the worry‐ and rumination‐driven processes that interfere with sleep. Within the PCT framework (Flett et al. 2015), perfectionistic tendencies are understood to stem primarily from self‐evaluative and socially evaluative concerns. This conceptualisation aligns well with the widely used distinction between perfectionistic strivings and perfectionistic concerns proposed by Stoeber and Otto (2006), which have been shown to differentially relate to cognitive processes. Perfectionistic concerns reflect worries about the negative consequences of imperfection, whereas perfectionistic strivings describe the pursuit of excessively high personal standards. Importantly, these two dimensions are thought to influence sleep through distinct pathways. According to Lundh and Broman (2000), perfectionistic strivings may promote maladaptive interpretations of sleep, for example by fostering unrealistic expectations and low tolerance for nights of suboptimal sleep. Perfectionistic concerns, on the other hand, are more closely tied to processes that directly interfere with sleep, such as increased worry, rumination in reaction to unsuccessful attempts to fall asleep, and stronger negative emotional responses to daily setbacks. While perfectionistic concerns have been consistently linked to poor sleep and insomnia, perfectionistic strivings have shown weaker or inconsistent associations (Stricker et al. 2023). We argue that perfectionistic concerns may be particularly detrimental for sleep because they are characterised by heightened worry and ruminative thinking, aligning closely with the mechanisms described in cognitive insomnia models (Espie 2007; Harvey 2002).

Besides an interindividual vulnerability for poor sleep, sleep in students is also shaped by environmental and time‐varying factors, most notably stress. Stress and sleep are closely linked, sharing common neural pathways and neurochemical processes (Lo Martire et al. 2020). University students face various potential stressors, including intense academic and social demands, often coupled with financial pressures (Owens et al. 2017; Peltz et al. 2021; Wang and Bíró 2021). Accordingly, 53% of 18,000 students in a German study reported high levels of stress (Herbst et al. 2016), making students a high‐risk group for stress‐related diseases, not least sleep disturbances. A meta‐analysis in undergraduate students found moderate associations between stress and sleep quality, although most of the evidence has been obtained through cross‐sectional studies (Gardani et al. 2022). Micro‐longitudinal studies that track daily fluctuations in both stress and sleep ‐ an essential approach given the pronounced intraindividual variability in students' sleep (Phillips et al. 2017) ‐ are far less common and have yielded mixed results in different populations. While some studies stated that stress during the day predicted reduced objective sleep time (Schmidt et al. 2024; Slavish et al. 2021; Yap et al. 2020), others reported no association with subjective or objective sleep parameters (Hanson and Chen 2010; Maher et al. 2022; Sin et al. 2017).

The PCT may help explain these mixed findings by suggesting that individuals high in perfectionistic concerns are likely to experience stress‐induced increases in cognitive activation (Flett et al. 2015), which in turn could negatively affect sleep. Students may be especially vulnerable to this pattern because a substantial portion of the stressors they encounter is evaluative in nature, such as performance‐based assessments, competitive academic environments, and socially comparative contexts. Such socially evaluative demands directly challenge core self‐standards, heighten concerns about external judgement, and reliably elicit stronger physiological stress responses (Dickerson and Kemeny 2004; Gruenewald et al. 2004). Accordingly, cortisol levels of students were shown to be higher during the academic term than during summer break (Stetler and Guinn 2020). We hypothesise that the relationship between daily stress and students' sleep is moderated by perfectionistic concerns, such that higher perfectionistic concerns strengthen the negative association between daily stress and sleep. Empirical evidence for this assumption is mixed: Johann et al. (2017) observed a more pronounced association between perfectionism and sleep during the first laboratory night of their polysomnographic study, which they interpreted as reflecting an interaction with stress. In contrast, Molnar et al. (2020) did not find evidence for such moderating effects of perfectionism in cross‐sectional samples of undergraduate students and adults. However, their design did not allow examination of within‐person variation in sleep parameters even though potential interaction effects between perfectionism and stress may be most evident at the day‐to‐day level, where fluctuations in evaluative demands and corresponding cognitive reactions typically unfold (Russell and Anderson 2019). Moreover, the precise pathways through which these factors may (jointly) affect sleep remain insufficiently understood. We argue that both stress and perfectionistic concerns may impair sleep through heightened cognitive activity prior to sleep.

Cognitive pre‐sleep arousal reflects the mental state immediately preceding sleep, characterised by heightened mental alertness including dysfunctional cognitions such as worry and rumination (Nicassio et al. 1985). These cognitively activating processes are well‐established impediments to initiating and maintaining sleep (Espie 2023; Lemyre et al. 2020). Given the persistent cognitive reactivity to stress and evaluative threat assumed in the PCT (Flett et al. 2015), individuals who experience high levels of stress and exhibit strong perfectionistic concerns may carry these intrusive thought processes into the pre‐sleep period, resulting in elevated cognitive pre‐sleep arousal. Accordingly, cognitive pre‐sleep arousal may represent the proximal mechanism through which perfectionistic cognitive tendencies ‐ either in combination with or triggered by daily stress ‐ translate into poorer sleep outcomes. In line with this assumption, cognitive pre‐sleep arousal has been identified as a mediator in the relationship between daily stress and sleep disturbances (Tousignant et al. 2019; Winzeler et al. 2014) and dysfunctional cognitions have been implicated in the link between perfectionism and sleep (Akram et al. 2020; Lin et al. 2019). To date, only the study by Küskens et al. (2024) has simultaneously examined perfectionism, stress, and pre‐sleep arousal, finding that pre‐sleep arousal, rather than perfectionism or stress, showed a robust association with poor sleep and interacted with perfectionistic concerns to predict self‐reported sleep parameters (Küskens et al. 2024). However, their study focused exclusively on individuals with clinically diagnosed insomnia, which limits the generalisability of their findings to non‐clinical populations. In addition, because cognitive pre‐sleep arousal reflects the mental state during the transition to sleep and is therefore temporally proximal to actual sleep, we conceptualise it ‐ unlike Küskens et al. (2024) ‐ as a mediating mechanism through which stress and perfectionistic concerns influence sleep.

**FIGURE 1:**
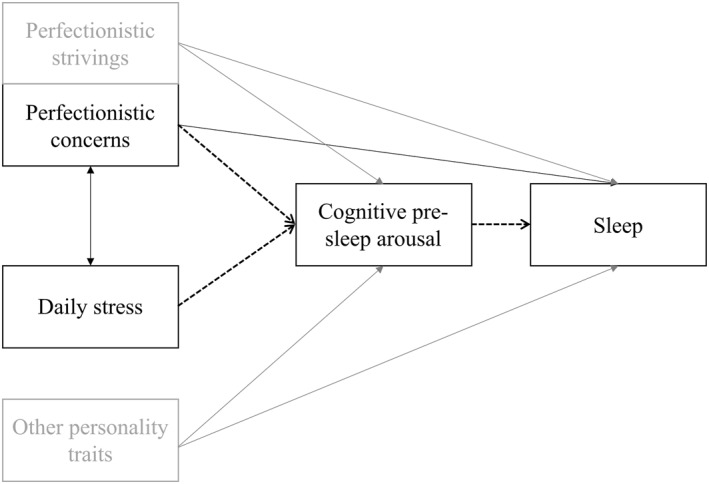
Conceptual model. Solid lines indicate the direct effects tested in the multilevel models. Dashed lines indicate the indirect (mediation) pathways tested in the multilevel structural equation models. Black lines show associations included in our primary hypotheses while gray lines show associations of covariates. Gray boxes represent covariates; “Other personality traits” include the Big Five personality dimensions and emotional distress, while sex was included as an additional covariate (not shown).

Overall, we argue that perfectionism in the form of perfectionistic concerns functions as a cognitive‐dispositional vulnerability that not only directly impairs sleep but also heightens cognitive reactivity to daily stressors, thereby increasing pre‐sleep arousal and further contributing to poorer sleep. Specifically, we investigate in a student sample whether (1) perfectionistic concerns and daily stress are associated with poorer subjective and objective sleep, (2) perfectionistic concerns exacerbate the within‐person association between daily stress and sleep, such that stressful days are particularly detrimental for individuals high in perfectionistic concern, and (3) cognitive pre‐sleep arousal arises from stress as well as perfectionistic concerns and, in turn, mediates their impact on sleep (see conceptual model in Figure 1). By applying a micro‐longitudinal design with frequent repeated assessments which is ideally suited for detecting within‐person processes (Simor et al. 2015), this study aims to extend prior findings from clinical samples, to advance theoretical models of stress–sleep interactions, and to identify potentially modifiable processes that may inform tailored interventions for improving sleep health in students.

## 2 Method

The study was part of a broader project that covered additional constructs in the context of sleep (e.g., bedtime procrastination and physical activity) beyond those mentioned in this study. After obtaining ethical approval from the Ethics Commission of the Faculty of Behavioural and Cultural Studies at Heidelberg University (protocol number: AZ Schm 2023 1/1), methods and statistical analyses were preregistered at the Open Science Framework (OSF; registration DOI: https://doi.org/10.17605/OSF.IO/3FXYP; for data and code, see: https://osf.io/p3yvn/?view_only=f7aea45c727e44fe8effa561031eac1f). All participants provided informed consent prior to enrolment.

### 2.1 Recruitment and Sample

Participants were recruited through lectures and seminars at the University of Heidelberg, as well as through online media. The study was advertised as a sleep study for students, aiming to track sleep as well as related factors and was rewarded with course credits. Eligible participants were university students aged 18 years or older. Individuals were excluded if they worked night or rotating shifts, lived with children under the age of four, used illegal drugs regularly, took psychotropic or sleep medication, or were currently receiving treatment for an acute sleep or mental health disorder. Conducting a power analysis with the software G*Power 3.1.9.2 (Faul et al. 2009) resulted in a necessary sample size of 75 students to detect medium sized effects of *f* ^*2*^ = 0.20 with *α* = 0.05 and power = 0.90 in regression analyses with three predictors (perfectionistic concerns, perfectionistic strivings, and stress). This sample size was used as guidance and aligns with simulation studies which specify a minimum sample size of 50 level‐two units for nested data (Maas and Hox 2005).

### 2.2 Procedure

The survey was conducted between December 2023 and February 2024 (covering the period from the middle to the end of the semester) after piloting the questionnaires and procedures with 16 participants. All questionnaires were in German language and administered via SoSci Survey (www.soscysurvey.de; Leiner 2024). Participants were given access to a screening questionnaire for exclusion criteria via link or QR code. Three days before their respective study start, an initial questionnaire assessing demographic variables and trait constructs was sent out. This needed to be completed by the participant's starting date, at which a commercially available wearable (Fitbit Alta HR) was provided to them for the next 14 days. Fitbit devices have shown convincing accuracy (between 0.81 and 0.91) in detecting sleep periods compared to polysomnography (Haghayegh et al. 2019). Each participant was guided through the setup of the Fitbit and its connection with their smartphone to ensure compliance with the study protocol. Participants were instructed not to use any optional or social features of the Fitbit device (e.g., activity goals, messaging, or any reminders). At the end of the study, they were asked whether they used additional features, serving as a double‐check to further ensure compliance with these settings. Consistent with a micro‐longitudinal study design, a link to a daily diary questionnaire was sent out to the participants each morning at 5 a.m. with a reminder at 10 a.m. over the following 14 days. On the last day of participation, after filling out the last daily diary, a link to a final questionnaire was provided, which contained questions about the study period (e.g., critical events during study period).

### 2.3 Measures

#### 2.3.1 Initial Questionnaire

The initial questionnaire included demographic variables and trait constructs.

##### 2.3.1.1 Sociodemographic Variables

Age, gender, study subject, intended degree, relationship status, and living situation of the participants were assessed. Additionally, to measure general sleep quality, the German translation of the PSQI (Buysse et al. 1989; Hinz et al. 2017) was implemented. The scale consists of 18 self‐assessment questions for the quantitative assessment of sleep quality, with a total score between 0 and 21. Total scores above 5 are interpreted as indicating bad sleepers. In our study, the PSQI showed a low internal consistency with a Cronbach's *α* of 0.57. Furthermore, the subjectively perceived stress experience of students was measured with the Heidelberger Stress‐Index (HEI‐STRESS; Schmidt and Obergfell 2011). The HEI‐STRESS consists of three items with different response scales. The answer for the first item (“Based on the last four weeks: How stressed did your studies make you feel?”) is indicated on a scale from 0 (*not stressed at all*) to 100 (*completely stressed*). The second item (“How often have you felt ‘stressed and tense’ in the last 4 weeks?”) is answered on a scale from 1 (*never*) to 5 (*daily*). And for the third item (“How would you describe your life at the moment? As…”) participants can describe their life as 1 (*not stressful at all*), 2 (*somewhat stressful*), 3 (*moderately stressful*), 4 (*quite stressful*), or 5 (*very stressful*). Responses were rescaled to a common 0 ‐ 100 metric, summed, and divided by three to yield a composite score, with higher values indicating greater perceived stress. The HEI‐STRESS showed an acceptable internal consistency with Cronbach's *α* of 0.79.

##### 2.3.1.2 Multi‐Dimensional Perfectionism

The two dimensions of perfectionism, perfectionistic concerns and perfectionistic strivings, were measured with the German translation of the Frost Multidimensional Perfectionism Scale (FMPS; Stöber 1995). Consistent with previous research and factorial analyses (Cox et al. 2002; Smith, 2019; Stricker et al. 2022), a combination of the FMPS subscales *concern over mistakes* (nine items) and *doubts about actions* (4 items) were used to capture perfectionistic concerns. An example item for *concern over mistakes* is “If I fail at work/school, I am a failure as a person”, and for *doubts about actions*, “Even when I do something very carefully, I often feel that it is not quite right”. Perfectionistic strivings were measured with the FMPS subscale *personal standards*, consisting of seven items. An example item is “If I do not set the highest standards for myself, I am likely to end up a second‐rate person”. Items were scored on a 5‐point Likert scale from 1 (*does not apply at all*) to 5 (*applies exactly*). For both perfectionistic strivings and perfectionistic concerns, item responses were summed to create composite scores, with higher scores indicating higher levels of the respective dimension. Cronbach's alpha was 0.88 for perfectionistic concerns (indicating excellent internal consistency) and 0.78 for perfectionistic strivings (reflecting acceptable reliability).

##### 2.3.1.3 Control Variables

Emotional distress was measured with the German translation of the ultra‐brief version of the Patient Health Questionnaire (PHQ‐4; Kroenke et al. 2009; Löwe et al. 2010). The PHQ‐4 consists of four items regarding core criteria for anxiety and depression disorders, introduced by the question “Over the last 2 weeks, how often have you been bothered by the following problems?”. Participants then indicate their answer for each item on a 4‐point Likert scale from 0 (*not at all*) to 3 (*nearly every day*). Item scores were summed to form a composite score (0–12). The PHQ‐4 showed good internal consistency with Cronbach's *α* of 0.83. To measure the dimensions of the five‐factor model of personality, the Big‐Five‐Inventory‐10 (BFI‐10; Rammstedt and John 2007) was used. Each of the ten items starts with “I see myself as someone who…” and is answered on a 5‐point Likert scale from 1 (*does not apply at all*) to 5 (*applies exactly*). Two items per trait were averaged to compute scores for neuroticism, extraversion, openness, agreeableness, and conscientiousness.

#### 2.3.2 Daily Diary Measures

The daily questionnaire included measures of subjective stress as well as of subjective and objective sleep.

##### 2.3.2.1 Sleep

Subjective sleep quality was measured with two items adapted from the Pittsburgh Sleep Diary (Monk et al. 1994), namely “Subjectively, how well did you sleep?” and “How rested did you feel when you woke up?”. Both items were answered on a scale from 0 (*very bad*/*not at all*) to 100 (*very good*/*very rested*) and were averaged for a total score. As recommended by Eisinga et al. (2013), the Spearman‐Brown coefficient was calculated as an indicator for the reliability of two‐item scales, which was 0.80 in our study. Additionally, the subjective SOL was assessed with the item “How long did it take you to fall asleep?”. For objective recording of sleep duration, participants were asked to transfer the indication on sleep duration displayed in the Fitbit Alta HR app (EU certification: Directive 2014/53/EU) to the daily questionnaire. To minimise transmission errors, specific instructions were provided in the questionnaire on how to find the measured sleep duration in the Fitbit app. This procedure was chosen for data privacy reasons, as participants used their personal Fitbit and Google accounts, to which the study team did not have access, and because the device's limited memory capacity prevented a reliable direct download of data over the entire study period. According to the manufacturer's information, the Fitbit Alta HR uses heartrate and body movements to assess sleep, whereby the sleep duration is already adjusted for awake/restless phases after falling asleep (Kollat 2022). If there were problems with sleep recording, participants could give an estimate of their sleep duration, sleep time, and wake‐up time.

##### 2.3.2.2 Daily Stress

To capture stress from the previous day, participants answered the question “How stressed did you feel yesterday?” on a scale from 0 (*not at all*) to 100 (*very stressed*) based on the HEI‐STRESS (Schmidt and Obergfell 2011).

##### 2.3.2.3 Cognitive Pre‐Sleep Arousal

Cognitive pre‐sleep arousal was assessed using the Cognitive Arousal subscale of the validated German version of the Pre‐Sleep Arousal Scale (Gieselmann et al. 2012). This subscale consists of 8 items that capture cognitive activity experienced prior to sleep onset (e.g., worry, intrusive thoughts, mental overactivation), which are commonly associated with sleep disturbances. Participants rated how intensely they typically experienced each item during the period just before falling asleep, using a 5‐point Likert scale ranging from 1 (not at all) to 5 (extremely). Composite scores were calculated by averaging the items, with higher values indicating greater cognitive pre‐sleep arousal. The scale showed an excellent reliability with a Cronbach's alpha of 0.91.

##### 2.3.2.4 Control Variables

To exclude days of drug‐induced sleep abnormalities, participants were asked if they used sleep medication, drank alcohol, or consumed cannabis or other illegal drugs in each daily questionnaire.

#### 2.3.3 Final Questionnaire Measures

The final questionnaire collected information about the participants' study period. Once again, screening criteria such as medical/therapeutic treatment for sleep problems or shift work were checked. Additionally, participants were asked if they experienced a critical life event during study period and, if so, how strongly this affected their stress and sleep behaviour (rating on a scale from 0 to 100).

### 2.4 Statistical Analysis

All analyses were conducted in the open‐source statistical programme R (Version 4.5.1; R Core Team 2021). Correlations were calculated using the R package *correlation* (Makowski et al. 2020) with Spearman rank‐order correlation coefficients calculated for correlations of sex with all other study variables due to its dichotomous nature. All other bivariate associations between the variables were examined using Pearson's product‐moment correlation coefficient. Data was analysed using multilevel modelling to account for the nested structure of measured days within individuals. For implementation, the R packages *lme4* and *lmerTest* were used (Bates et al. 2015; Kuznetsova et al. 2017). While stress was group‐mean centred for multilevel analyses, all person‐level variables (except of sex) were grand‐mean centred. All analyses were conducted using all available data. Due to the high diary compliance (see results), missing data were not imputed. Cases with missing values for a specific outcome were excluded listwise for that analysis.

#### 2.4.1 Multilevel Models

First, requirements for multilevel models were checked. Since the residuals of subjective SOL turned out to be right‐skewed, the variable was transformed using the square root transformation. Violations of the homoscedasticity assumption existed for subjective sleep quality and SOL. However, results of multilevel analyses are largely robust to those violations (Schielzeth et al. 2020), so no further transformations were performed. In addition, the Rainbow test indicated a statistically significant deviation from linearity for multilevel models of subjective root‐transformed SOL which can lead to a heightened error component in the residuals and biased estimates (Gorard 2003). However, the graphical inspection revealed no meaningful non‐linear pattern, suggesting that any departure from linearity may be minor.

Up to four multilevel models were calculated for each outcome to test the influence of stress, multi‐dimensional perfectionism, and covariates, as well as a possible cross‐level interaction between the predictors, on the objective sleep duration, subjective sleep quality, and subjective SOL. First, the intercept‐only model was calculated to determine whether significant between‐person and within‐person variation existed in the sleep parameters. The second model (random‐intercept‐fixed‐slope model) included all control variables (sex, emotional distress, neuroticism, extraversion, openness, conscientiousness, agreeableness), the person‐level predictors perfectionistic concerns and perfectionistic strivings, and the within‐person component of daily stress. Thereafter, random slopes of daily stress were modulated in the third model (random‐intercept‐random‐slope model). Only if this model showed a significant variance in the stress‐sleep slope, the fourth model including the cross‐level interaction was set up. In this model, the person‐level predictors perfectionistic concerns and perfectionistic strivings are modulated as predictors of the intercept and the stress‐sleep slope. This latter effect represents the moderating role of perfectionistic concerns or perfectionistic strivings on the stress‐sleep association. All models were fitted using the maximum likelihood estimation method. Improvements in fit between the models were evaluated using likelihood‐ratio tests with the R package *lmtest* (Zeileis and Hothorn 2002) and effect sizes of the multilevel models were determined using Nakagawa's *R* ^*2*^ (Nakagawa et al. 2017), which was calculated with the R package *performance* (Lüdecke et al. 2021). Variance explained by fixed effects (*marginal R* ^*2*^) as well as by fixed and random effects (*conditional R* ^*2*^) are reported.

#### 2.4.2 Mediation Models

Multilevel structural equation modelling was conducted using the *lavaan* package in R (Rosseel 2012) to test whether cognitive pre‐sleep arousal mediated the association between daily stress, perfectionistic concerns, and sleep outcomes (objective sleep duration, subjective sleep quality, and subjective sleep latency). Separate mediation pathways were specified at the within‐person (level 1) and between‐person (level 2) levels; thus, 1–1‐1 mediation models were implemented. Model parameters were estimated using maximum likelihood estimation. The same control variables used for the multilevel models were included at the between‐person level. Mediation models were specified only for predictor variables that fulfiled the basic empirical criteria for mediation. Specifically, significant bivariate associations between the independent variable and the mediator (cognitive pre‐sleep arousal), as well as between the mediator and the sleep outcome had to be present. Further exploratory mediation analyses were conducted when a covariate showed significant associations in the multilevel models with both cognitive pre‐sleep arousal and a sleep variable; this was the case for emotional distress. The usual inference statistics (chi‐square test) in terms of the quality of fit are reported. However, as these are partly oversensitive, the model fit of the mediation models was considered acceptable if the comparative fit index (*CFI*) and Tucker‐Lewis index (*TLI*) were greater than 0.95 (Xia and Yang 2019). Additionally, the root mean square error of approximation (*RMSEA*) should be less than 0.06, and the standardized mean square residual (*SRMR*) should be less than 0.08 (Hu and Bentler 1999; Xia and Yang 2019).

#### 2.4.3 Justification for Covariates

Sex was included as control variable since studies revealed significant sex differences in sleep parameters (Putilov et al. 2021; Reyner and Horne 1995; Tsai and Li 2004). In addition, previous research has shown that some broader personality constructs are linked to sleep outcomes and should thus be controlled in order to isolate the additional predictive value of perfectionism dimensions. For example, with respect to well‐established personality dimensions like the five‐factor model of personality (McCrae and Costa 1987), Stricker et al. (2022) discussed that perfectionistic strivings may share features with conscientiousness and extraversion, traits linked to better sleep, while on the other hand they also overlap with neuroticism, which is associated with poorer sleep (Slavish et al. 2018; Stephan et al. 2018). We included the full set of Big Five dimensions to account for shared variance across the broader personality system. Additionally, emotional distress, which encompasses feelings of anxiety and depression, represents another well‐documented determinant for sleep (Cunningham et al. 2015; Seixas et al. 2015) and was considered as a relevant covariate.

#### 2.4.4 Sensitivity Analyses

Sensitivity analyses were carried out by testing the postulated multilevel models with and without days of alcohol or cannabis consumption, or by excluding participants who indicated a critical life event in the final questionnaire which effected their sleep behaviour drastically (rating of 70 or higher out of 100). Further sensitivity analyses extended the multilevel models by adding theoretically relevant covariates, including the between‐person component of daily stress (average stress), the previous night value of the respective outcome (lag‐1), and a dummy‐coded indicator for weekend days (0 = weekdays, 1 = weekend days).

## 3 Results

### 3.1 Study Population

In total, 94 students participated in the study. Due to medical/therapeutic treatment (*n* = 5) and the regular consumption of cannabis (*n* = 1) six participants had to be excluded. No participants were excluded due to insufficient data, defined as fewer than four completed daily diaries per week. Therefore, the final sample consisted of *N* = 88 university students. The age ranged from 18 to 44 years (*M* = 22.47, SD = 3.48), with *n* = 66 (75%) being female. Sixty‐seven (76.1%) participants studied psychology, *n* = 11 (12.5%) sports science and *n* = 10 (8.8%) other disciplines, while *n* = 64 (72.7%) of the total sample were undergraduates. Regarding relationship status and living situation, *n* = 38 (43.2%) indicated being single and *n* = 14 (15.9%) living alone. Table 1 shows descriptive statistics of all study variables. Overall, sleep quality assessed at the beginning of the study using the PSQI indicated that more participants reported poor or clinically relevant sleep disturbances (Global PSQI score ≥ 6) than healthy sleep patterns. Perceived stress levels among students showed considerable variation, ranging from low to very high levels. On average, participants slept just over 7 hours per night, rated their sleep quality as moderate, and reported a subjective sleep onset latency of around 20 min. In terms of perfectionism, participants showed moderate levels of both perfectionistic concerns and perfectionistic strivings. Daily stress ratings also showed a wide range, with an average indicating moderate stress levels. We exploratively assessed sex differences on all outcome variables as well as perfectionism dimensions, emotional distress, and daily stress. Women reported longer sleep duration (*M* = 433.2 vs. 415.1 min, *p* = 0.021), lower subjective sleep quality (*M* = 63.4 vs. 69.7, *p* = 0.026), longer SOL (*M* = 22.1 vs. 13.0 min, *p* < 0.001), and higher stress (*M* = 50.0 vs. 38.9, *p* = 0.007) compared to men.

**TABLE 1:** Descriptive statistics of study variables.

Variables	*N*	Response scale	Minimum	Maximum	*M*	SD	*ICC*
Objective sleep duration	1182	hours	2.63	13.17	7.15	1.06	0.18
Subjective sleep quality	1206	0–100	6	100	65.06	18.04	0.26
Subjective SOL	1202	minutes	0	180	19.51	19.94	0.25
Cognitive pre‐sleep arousal	1206	1–5	7	33	11.99	5.52	0.40
Daily stress	1206	0–100	0	100	47.09	24.77	
Perfectionistic concerns	88	1–5	1.15	4.77	2.41	0.74	
Perfectionistic strivings	88	1–5	1.57	4.86	3.15	0.70	
Emotional distress	88	0–3	0	3	0.74	0.63	
Neuroticism	88	1–5	1	5	3.10	1.01	
Extraversion	88	1–5	1	5	3.53	1.01	
Openness	88	1–5	1.5	5	3.70	0.93	
Agreeableness	88	1–5	1.5	5	3.57	0.84	
Conscientiousness	88	1–5	2.5	5	3.82	0.66	

*Note:* Sample sizes, response scales, minimums, maximums, means, standard deviations, and intraclass correlations of all study variables are displayed.

Abbreviations: ICC, Intraclass correlation; SOL, Sleep onset latency.

### 3.2 Compliance

Of the 1232 possible diary entries, 26 nights were not analysed, with 14 entries being completely missing and 12 excluded due to the use of sleep medication during the night, incomplete completion of the daily questionnaire, or retrospective completion more than 24 h after initial receipt. This resulted in 1206 valid diary reports, reflecting a high compliance rate of 97.89%. Due to recording errors, the objective measurement of sleep duration was excluded for 24 nights. This was assumed if fitbit‐recorded sleep duration and participants' subjective estimates deviated by more than 120 min, which removed only a few extreme outliers while retaining the vast majority of nights within a plausible range of agreement. SOLs of more than 3 hours were excluded, affecting four nights. Therefore, while all 1206 nights were included in the analyses of subjective sleep quality, analyses of objective sleep quality were based on 1182 daily diary reports, and analyses of subjective SOL were based on 1202 reports. No participant indicated active use of any Fitbit features intended to enhance sleep.

### 3.3 Intercorrelations Between Study Variables

Table 2 displays the bivariate correlations between all study variables at day‐ and person‐level. While all sleep parameters were intercorrelated at the day‐level, only subjective sleep quality and subjective SOL were correlated at the person‐level. None of the perfectionism dimensions was correlated with any sleep parameter. However, they were positively associated with each other and with daily stress. Daily stress was positively associated with subjective SOL at the person‐level and negatively associated with objective sleep duration and subjective sleep quality at the day‐level. Cognitive pre‐sleep arousal was significantly associated with all sleep parameters as well as with perfectionistic concerns. Sex was significantly associated with all sleep parameters, indicating that women slept longer but rated their sleep quality worse and had a longer subjective SOL than men in this sample.

**TABLE 2:** Intercorrelations between study variables.

Variables	1.	2.	3.	4.	5.	6.	7.	8.	9.	10.	11.	12.	13.	14.
1. Objective sleep duration		0.10	0.17	−0.19	−0.16	−0.05	−0.07	−0.22^*^	0.01	0.09	0.05	0.02	−0.06	−0.25^*^
2. Subjective sleep quality	0.40^***^		−0.23^*^	−0.17	−0.25^*^	−0.06	0.13	−0.15	−0.24^*^	0.01	−0.03	−0.05	0.11	0.27^*^
3. Subjective SOL	−0.13^***^	−0.30^***^		0.23^*^	0.34^**^	0.04	0.07	−0.01	0.18	0.09	0.04	0.06	−0.02	−0.38^***^
4. Daily stress	−0.06^*^	−0.10^***^	0.05		0.61^***^	0.43^***^	0.32^**^	0.38^***^	0.45^***^	−0.11	0.08	−0.18	0.21^*^	−0.28^**^
5. Cognitive pre‐sleep arousal	−0.20^***^	−0.36^***^	0.45^***^	0.25^***^		0.47^***^	0.15	0.54^***^	0.58^***^	−0.18	−0.17	0.04	0.02	−0.23^*^
6. Perfectionistic concerns							0.46^***^	0.44^***^	0.63^***^	−0.26^*^	−0.04	−0.19	0.01	−0.10
7. Perfectionistic strivings								0.10	0.18	−0.02	0.00	−0.14	0.32^**^	0.11
8. Emotional distress									0.60^***^	−0.10	−0.09	−0.18	0.02	−0.06
9. Neuroticism										−0.15	−0.15	−0.06	0.05	−0.35^***^
10. Extraversion											0.15	0.21^*^	0.29^**^	−0.17
11. Openness												−0.04	0.00	0.04
12. Agreeableness													0.11	−0.23^*^
13. Conscientiousness														−0.10
14. Sex														

*Note:* Correlations below diagonal present day‐level correlations. Correlations above diagonal represent person‐level correlations. While sex was coded as 1 for women and 2 for men, higher values on the remaining variables indicate a stronger expression of the property. Correlations regarding sex were calculated using Spearman‐correlation method due to its dichotomous nature. All other correlations were calculated using Pearson‐correlation method.

Abbreviation: SOL = Sleep onset latency.

^*^ *p* < .05.

^**^ *p* < .01.

^***^ *p* < .001.

### 3.4 Multilevel Analyses

Results of the final models of the multilevel analyses are presented in Table 3 and stepwise models are provided in the Supplementary Information 1. In all models, daily stress was specified as a within‐person predictor, whereas all other predictors were entered at the between‐person level. Results of sensitivity analyses without days with alcohol or cannabis use (*N* = 88, 1076 days), without participants who reported a critical life event (*N* = 84, 1154 days), and with additional covariates (average stress, lag‐1 outcome, weekend) can be found in Supplementary Information 2.

**TABLE 3:** Results of the final models predicting the daily sleep outcomes.

Variables	Objective sleep duration		Subjective sleep quality		Subjective SOL		Cognitive pre‐sleep arousal	
	*B*	SE	*b*	SE	*b*	SE	*B*	SE
Fixed effects								
Intercept	451.40***	(10.92)	58.64***	(3.54)	5.14***	(0.36)	12.37***	(1.04)
Daily stress	−0.21*	(0.10)	−0.09**	(0.03)	0.01*	(0.00)	0.06***	(0.01)
Perfectionistic concerns	0.00	(0.49)	0.09	(0.16)	−0.02	(0.02)	0.06	(0.05)
Perfectionistic strivings	−0.25	(0.79)	0.16	(0.26)	0.04	(0.03)	0.02	(0.07)
Emotional distress	−4.04*	(1.62)	−0.23	(0.52)	−0.06	(0.05)	0.49**	(0.15)
Neuroticism	4.24	(4.95)	−2.17	(1.61)	0.21	(0.16)	0.91	(0.47)
Extraversion	2.44	(3.51)	0.09	(1.14)	0.02	(0.12)	−0.37	(0.33)
Openness	0.94	(3.43)	−0.94	(1.11)	0.08	(0.11)	−0.30	(0.33)
Agreeableness	−4.42	(4.06)	0.03	(1.32)	−0.01	(0.13)	0.67	(0.39)
Conscientiousness	−4.09	(5.31)	1.73	(1.72)	−0.15	(0.17)	0.00	(0.50)
Sex	−18.06*	(8.38)	5.12	(2.72)	−0.86**	(0.28)	−0.31	(0.80)
Random variances								
Intercept σμ0	590.10		71.62		0.74		6.34	
Daily stress σμ1			0.02					
Residual σε	3328.80		228.89		2.21		17.00	
Conditional *R* ^*2*^	0.18		0.29		0.30		0.44	
Marginal *R* ^*2*^	0.04		0.05		0.07		0.23	

*Note:* *N* = 88, 1182 days for objective sleep duration, 1206 days for subjective sleep quality, 1202 days for subjective sleep onset latency (SOL), and 1206 days for cognitive pre‐sleep arousal. SOL was transformed by square root transformation. Unstandardised estimates are displayed with standard errors given in parentheses. Sex, emotional distress, neuroticism, extraversion, openness, agreeableness and conscientiousness were entered as control variables. While sex was coded as 1 for women and 2 for men, higher values on the remaining variables indicate a stronger expression of the property.

^*^ *p* < .05.

^**^ *p* < .01.

^***^ *p* < .001.

#### 3.4.1 Objective Sleep Duration

A random‐intercept fixed‐slope model including the predictors perfectionistic concerns, perfectionistic strivings, daily stress, and all control variables showed a significant improvement in model fit over the intercept‐only model (χ^2^(10) = 18.55, *p* = 0.046). Allowing random slopes for daily stress did not further improve model fit (χ^2^(2) = 0.69, *p* = 0.709), indicating that participants responded similarly to fluctuations in daily stress in terms of their objective sleep duration. Consequently, no cross‐level interactions between daily stress and perfectionistic concerns were modeled. Within the random‐intercept fixed‐slope model, besides emotional distress and sex, daily stress (*b* = −0.21, Standard Error (SE) = 0.10, *p* = 0.033) emerged as significant predictor, indicating that participants slept less on stressful days. According to Nakagawa's *R* ^2^, 4% of the variance in sleep duration was explained by fixed effects, and 18% by the full model including random effects. Sensitivity analyses showed that excluding days with alcohol or cannabis use did not change the model structure, though the effect of daily stress on sleep duration became marginally significant (*p* = 0.057). Excluding participants who reported a critical life event led to only a marginal improvement in model fit of the random‐intercept fixed‐slope model (χ^2^(10) = 17.03, *p* = 0.074), with daily stress emerging as marginally significant predictor (*p* = 0.074). In the model using additional covariates, there was only a trend for a significant association of daily stress (*b* = −0.18, SE = 0.10, *p* = 0.084), but a significant association of average stress with shorter objective sleep duration (*b* = −0.48, SE = 0.23, *p* = 0.036).

#### 3.4.2 Subjective Sleep Quality

The random‐intercept fixed‐slope model significantly improved model fit over the intercept‐only model (χ^2^(10) = 24.85, *p* = 0.006). Adding random slopes for daily stress further improved fit (χ^2^(2) = 7.22, *p* = 0.027), and thus, a cross‐level interaction model was tested, including perfectionistic concerns × stress and perfectionistic strivings × stress interactions. However, neither interaction was significant (perfectionistic concerns: *b* = 0.003, SE = 0.004, *p* = 0.386; perfectionistic strivings: *b* = −0.003, SE = 0.007, *p* = 0.639), and their inclusion did not improve model fit (*χ* ^2^(2) = 0.26, *p* = 0.607). The final model was therefore the random‐intercept random‐slope model without interactions. In this model, daily stress was the only significant predictor, with higher daily stress associated with lower subjective sleep quality (*b* = −0.09, SE = 0.03, *p* = 0.006). A small correlation between random intercepts and slopes (*r* = 0.10) suggests that the negative impact of stress on sleep quality was slightly weaker in individuals with higher average sleep quality. Nakagawa's *R* ^2^ indicated that 5% of the variance was explained by fixed effects and 29% by the full model. Sensitivity analyses showed that excluding days with alcohol or cannabis use led to no significant improvement from adding random slopes (χ^2^(2) = 4.13, *p* = 0.127); thus, the random‐intercept fixed‐slope model was adopted for that subset. Still, daily stress remained a significant predictor. Excluding participants who reported a critical life event yielded the same final model as in the full sample, with daily stress as the sole significant predictor. In the model using additional covariates, higher sleep quality on the previous night and weekend days were positively associated with subjective sleep quality (both *p* < 0.05).

#### 3.4.3 Subjective Sleep Onset Latency

Again, the random‐intercept fixed‐slope model significantly improved model fit over the intercept‐only model (χ^2^(10) = 21.99, *p* = 0.015). Adding a random slope for daily stress did not improve model fit (χ^2^(2) = 1.24, *p* = 0.539). In the final random‐intercept fixed‐slope model, daily stress was a significant predictor (*b* = 0.01, SE = 0.00, *p* = 0.046) for longer root‐transformed SOL, indicating that participants needed longer to fall asleep after stressful days. According to Nakagawa's *R* ^*2*^, 7% of the variance in subjective SOL was explained by fixed effects, while 30% was explained by the full model. Sensitivity analyses, excluding days with alcohol or cannabis use or participants who reported a critical life event affecting their sleep, confirmed the main findings: the random‐intercept fixed‐slope model remained the best‐fitting model, and daily stress was (besides sex) the only significant predictor for SOL. In the extended model including additional covariates, the association of daily stress was no longer significant (*b* = 0.00, SE = 0.00, *p* = 0.216), whereas average stress showed a trend for a significant association with longer SOL (*b* = 0.01, SE = 0.01, *p* = 0.074). Moreover, longer SOL on the previous night was associated with longer SOL the following day, whereas weekend days were associated with shorter SOL (both *p* < 0.01).

#### 3.4.4 Cognitive Pre‐Sleep Arousal

The random‐intercept fixed‐slope model significantly improved model fit compared to the intercept‐only model (χ^2^(10) = 123.46, *p* < 0.001). Although the random‐intercept random‐slope model showed a better fit (χ^2^(2) = 27.08, *p* < 0.001), the variance of the random slope for daily stress was close to zero and the model failed to converge. Therefore, the more parsimonious random‐intercept fixed‐slope model was retained. In the final random‐intercept fixed‐slope model, besides emotional distress, only daily stress was a significant predictor of stronger cognitive pre‐sleep arousal (*b* = 0.06, SE = 0.01, *p* < 0.001). Nakagawa's *R* ^2^ indicated that 23% of the variance in cognitive pre‐sleep arousal was explained by fixed effects and 44% by the full model. Sensitivity analyses using the fixed‐slope random‐intercept model confirmed a significant association of daily stress and emotional distress (in the analysis excluding individuals with a critical life event, also neuroticism) with cognitive pre‐sleep arousal. In the model with covariates, average stress was a significant predictor (*b* = 0.10, SE = 0.02, *p* < 0.001) alongside openness, agreeableness, previous‐day cognitive pre‐sleep arousal, and weekend days (all *p* < 0.05).

### 3.5 Mediation Analyses

**FIGURE 2:**
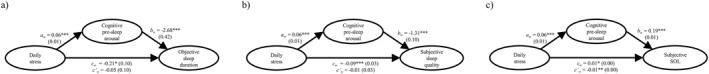
Multilevel structural equation modelling mediation model predicting (a) sleep duration, (b) sleep quality, and (c) root‐transformed sleep onset latency (SOL) at the within‐person‐level. *N* = 88, 1182 days for objective sleep duration, 1206 days for subjective sleep quality, 1202 days for subjective sleep onset latency (SOL). a_w_ = within‐person path from daily stress to cognitive pre‐sleep arousal; b_w_: within‐person path from cognitive pre‐sleep arousal to the respective sleep parameter; *c*_w_ = total within‐person effect; c'_w_ = direct within‐person effect. Unstandardised parameter estimates are listed with standard errors in parentheses. Sex, emotional distress, perfectionistic concerns, perfectionistic strivings, neuroticism, extraversion, openness, agreeableness, and conscientiousness were entered as control variables. For simplicity, control variables and residual variances are not displayed. *p < 0.05; **p < 0.01; ***p < 0.001.

Figure 2 illustrates the within‐person indirect effects for all three sleep outcomes, when controlling for between‐person effects, sex, emotional distress, and the Big Five traits. On days with higher‐than‐usual stress, participants reported significantly increased cognitive pre‐sleep arousal (a_w_ paths), which was in turn associated with all sleep parameters (b_w_ paths). The significant total within‐effects of daily stress on sleep in all models (c_w_ paths) were partly explained by mediation through cognitive pre‐sleep arousal, leading to shorter objective sleep duration (Panel a; indirect within‐person effect = −0.16, SE = 0.03, *p* < 0.001), lower sleep quality (Panel b; indirect within‐person effect = −0.08, SE = 0.01, *p* < 0.001), and longer subjective SOL (Panel c; indirect within‐person effect = 0.01, SE < 0.01, *p* < 0.001). At the between‐person level, the only significant indirect effect was from average daily stress to subjective SOL via cognitive pre‐sleep arousal (indirect between‐person effect = 0.21, SE = 0.07, *p* = 0.004; see Supplementary Information 3). The model fits produced mixed results for all three mediation models, meeting the predefined cut‐off criteria for RMSEA and SRMR_within_, but not for CFI, TLI, SRMR_between_ or the chi‐square test (see Supplementary Information 4). Exploratory adjustments, including the addition of paths suggested by modification indices, the specification of cognitive pre‐sleep arousal as a latent factor, or the inclusion of additional within‐person level covariates (i.e., weekend, lag‐1 outcome) did not yield substantial improvements in model fit.

In line with the analysis plan, mediation models were only estimated for predictor variables that showed a significant association with cognitive pre‐sleep. No such association was found for perfectionistic concerns, and thus, no mediation model was specified for this variable. In contrast, emotional distress was significantly associated with cognitive pre‐sleep arousal. Therefore, an exploratory mediation analysis was conducted to examine whether cognitive pre‐sleep arousal mediated the relationship between emotional distress and each of the three sleep outcomes. A significant indirect effect was observed for SOL (indirect between‐person effect = 0.09, SE = 0.03, *p* = 0.007; see Supplementary Information 5).

## 4 Discussion

This is the first study to investigate the relation of perfectionism, cognitive pre‐sleep arousal and stress, as well as their potential interactions, with different sleep parameters in students using a micro‐longitudinal design. Guided by the PCT, we hypothesised that perfectionistic concerns and daily stress would impair sleep because both factors are expected to heighten cognitive reactivity, and thereby increase pre‐sleep arousal. However, our results did not support this assumption: perfectionistic concerns were unrelated to sleep outcomes and showed no association with cognitive pre‐sleep arousal. Instead, daily stress emerged as the primary predictor, that was associated with an increase in cognitive pre‐sleep arousal and worse sleep the following night. Mediation analyses ‐ interpreted cautiously given suboptimal model fits ‐ suggest that cognitive pre‐sleep arousal may serve as a proximal mechanism linking daily stress to sleep parameters. Similarly, cognitive pre‐sleep arousal appeared to mediate the relationship between emotional distress and SOL. These findings remained robust across additional sensitivity analyses that excluded days with alcohol or cannabis use, as well as participants reporting major life events affecting their sleep. When additional covariates were included ‐ namely average stress, the lagged outcome variable, and weekend days ‐ average stress, rather than daily stress level, emerged as a stronger predictor of sleep duration and SOL. This suggests that chronic stress levels may exert a stronger influence on these outcomes than day‐to‐day fluctuations.

As expected, daily stress emerged as a predictor of objective sleep duration, subjective sleep quality, and SOL. This finding aligns with previous research demonstrating significant associations in both cross‐sectional (Almojali et al. 2017; Amaral et al. 2018; Lemma et al. 2012) and micro‐longitudinal designs (Åkerstedt et al. 2012; Schmidt et al. 2024; Slavish et al. 2021; Yap et al. 2020). The present study supports the idea that fluctuations in daily stress within university students are associated with changes in sleep outcomes, and not just the result of between‐person differences. According to the PSQI total score, the majority of participants had poor sleep quality, indicating problematic sleep behaviour in this sample. Therefore, daily stress may have become an additional factor that negatively affected their sleep. This could explain the discrepancy with other micro‐longitudinal studies that did not find an association between daily stress and sleep (Hanson and Chen 2010; Maher et al. 2022; Sin et al. 2017). For objective sleep duration, a 10‐point increase in daily stress (on a 0‐100 scale) was associated with a decrease of 2.10 min. This aligns with prior work showing similar small reductions in sleep duration per daily stress unit (Schmidt et al. 2024; Yap et al. 2020). The effect of daily stress on subjective sleep quality was also small (one‐point decrease in perceived sleep quality per 10‐point increase in stress), which may partly result from retrospective stress assessments covering the entire day. In contrast, Åkerstedt et al. (2012) reported stronger effects when assessing stress immediately before bedtime, suggesting that evening stress may be a more sensitive predictor of sleep quality. In this study, daily stress also predicted longer subjective SOL, contrary to previous micro‐longitudinal studies that found no such association (Slavish et al. 2021; Yap et al. 2020). Interestingly, an experimental study has shown that acute psychosocial stress can increase EEG‐measured SOL (Ackermann et al. 2019), indicating potential context‐ or population‐specific effects. Given the high levels of psychosocial and evaluative stress that university students often experience (Haruna et al. 2025; Olson et al. 2025; Stetler and Guinn 2020; Wang and Bíró 2021), they may represent a particularly vulnerable group for experiencing prolonged SOL. Including average stress and additional covariates in the multilevel models showed that chronic stress ‐ rather than daily stress ‐ was the more influential predictor of objective sleep duration (and showed a similar, though non‐significant, tendency for SOL). At the same time, the finding that daily stress remained the only significant predictor of subjective sleep quality suggests a dissociation between chronic and day‐to‐day stress effects. On the one hand, chronic stress may shift individuals' priorities away from sleep (thus reducing sleep duration) towards academic or task‐related activities. On the other hand, higher‐than‐usual stress on a specific day appears to impair perceived sleep quality irrespective of chronic stress levels.

The effect of stress on sleep in our study was found to be independent of perfectionism levels, with neither main nor interaction effects of perfectionistic concerns or strivings on any sleep parameters. While null findings for perfectionistic strivings align with prior evidence showing weak or non‐significant associations with poor sleep (Stricker et al. 2023), the lack of associations for perfectionistic concerns is more unexpected. The meta‐analysis by Stricker et al. (2023) found moderate correlations between perfectionistic concerns and global sleep quality, and consistent associations have been found with specific sleep parameters, including duration, quality, latency, disturbances, and daytime dysfunction (Azevedo et al. 2010, 2009; Johann et al. 2017; Lin et al. 2019; Molnar et al. 2020). In our data, perfectionistic concerns and perfectionistic strivings also showed no significant bivariate correlations with any sleep outcome. This suggests that the absence of effects is not due to confounding by third variables such as emotional distress or broader personality traits, but rather reflects a lack of association altogether. Perfectionism may play a stronger role in chronic sleep difficulties, whereas day‐to‐day variability is likely driven more by dynamic factors such as stress or coping (Brand et al. 2015). This would explain why daily diary studies in different populations have also failed to find reliable associations between perfectionism and subjective sleep or actigraphy‐derived sleep measures (Oh et al. 2024). Daily diary studies, particularly those including objective measures, are less prone to retrospective reporting bias or heightened expectations regarding sleep. Such tendencies may be especially common among perfectionistic individuals and could have inflated associations in cross‐sectional designs. From the perspective of the PCT, the results of this study suggest that the cognitive‐dispositional vulnerability associated with perfectionistic concerns does not necessarily translate into sleep‐impairing cognitive activity (Lemyre et al. 2020). Potentially, perfectionistic concerns may need to reach a higher threshold to meaningfully affect sleep. The relatively low and homogeneous levels observed in this non‐clinical student sample (*M* = 2.41, SD = 0.74 on a 1‐5 scale) may have not been sufficient to activate the cognitive‐emotional processes through which perfectionism was theorized to worsen subsequent sleep.

There was also no indication in this study that perfectionism moderates the effect of daily stress on sleep. Random slope models revealed no substantial slope variance for the daily‐stress‐sleep associations, and exploratory analyses confirmed that neither perfectionistic concerns nor strivings interacted with daily stress for any sleep parameter or improved the model fit. This was also reflected in sensitivity analyses additionally including average stress. The absence of an interaction between stress and perfectionism in predicting sleep outcomes in this study aligns with findings from a cross‐sectional study in undergraduate students (Molnar et al. 2020) and with recent results from the daily diary study by Küskens et al. (2024) among individuals with insomnia. Together with our finding of a main effect of daily stress, this indicates that the experienced stress may have elicited similar sleep‐relevant cognitive‐emotional reactions across individuals, leaving little room for trait‐based moderation. Future research should examine these cognitive and emotional responses to stress in detail to determine which ones impair sleep and to explain interindividual differences. In this regard, dysfunctional beliefs and attitudes about sleep have emerged as an important modifiable factor linking perfectionism with sleep (Akram et al. 2020; Chachos et al. 2023; Dautovich et al. 2021) but have not yet been explored in daily diary studies.

Results of this study suggest that cognitive pre‐sleep arousal could be a central mechanism affecting students' sleep. While perfectionistic concerns were not associated with pre‐sleep arousal, suggesting that perfectionism‐related worry may be down‐regulated before bedtime, daily stress seems to trigger perseverative cognitive activity that carries into the pre‐sleep period. In our study, daily stress significantly predicted cognitive pre‐sleep arousal in multilevel models. In the mediation models, cognitive pre‐sleep arousal significantly mediated the within‐person effect of daily stress on sleep duration, subjective sleep quality, and SOL. Given that several model fit indices indicated poor fit, these indirect effects must be interpreted with caution and should be regarded as exploratory rather than confirmatory. Nevertheless, the overall pattern aligns with earlier findings by Tousignant et al. (2019) and Winzeler et al. (2014) in samples from the general population and healthy young women, respectively. Our study extends these findings by confirming the mediating role of cognitive pre‐sleep arousal in a student population. Interestingly, Winzeler et al. (2014) did not find a mediating effect of cognitive arousal on actigraphy‐measured sleep efficiency. The divergent finding point to cognitive arousal processes as particularly important for the sleep of student populations. One possible explanation may be a perceived benefit of anticipatory thinking when facing academic tasks (Morsella et al. 2010) which may heighten cognitive activity at bedtime. However, this can only be clarified through studies that directly assess the content and temporal unfolding of bedtime cognitions.

The partially suboptimal model fit indices suggest that the proposed mediation models may not fully capture the complexity of the processes underlying sleep outcomes and that further theoretical refinement and empirical validation are needed. The intraclass correlations of the multilevel models for the sleep parameters indicate that the majority of variance in sleep parameters was within rather than between individuals. This imbalance restricts the capacity of the between‐person component of the mediation models to provide a good overall fit, suggesting that the suboptimal global fit is mainly driven by insufficiently modeled variance at Level 2 (i.e., all SRMR_between_ > 0.15) rather than by misrepresentation of daily within‐person dynamics. Correspondingly, exploratory analyses based on modification indices indicated that additional direct and indirect paths ‐ particularly from personality traits such as emotional distress and neuroticism to stress and cognitive pre‐sleep arousal ‐ may play a relevant role. These factors could act as upstream determinants of stress and cognitive activation. However, the inclusion of these exploratory paths did not lead to a model that met the predefined fit criteria and was therefore not retained for further analysis. Another possible explanation for the limited model fit is that the models only considered cognitive pre‐sleep arousal while somatic pre‐sleep arousal (i.e., physiological arousal such as elevated heart rate) is also linked to stress and sleep (Küskens et al. 2024; Tousignant et al. 2019; Winzeler et al. 2014).

Exploratory analyses revealed that, unlike perfectionism, emotional distress was significantly associated with objective sleep duration and cognitive pre‐sleep arousal. Psychological emotional distress has already been identified as a significant risk factor for impaired sleep quality and the onset of insomnia symptoms (Dressle and Riemann 2023; Seixas et al. 2015). A noteworthy finding is that, in our study, emotional distress did not (directly) predict the subjective measures of sleep quality or SOL. It is well known that objective measures of sleep do not always align with subjective reports (Benz et al. 2023). In this study, the discrepancy could reflect that objective sleep duration is more sensitive to behavioural shifts under emotional strain ‐ such as delayed bedtimes or early awakenings ‐ while subjective sleep quality represents a holistic appraisal that might be prone to state‐related influences. Moreover, the effect of emotional distress on subjective SOL appeared only indirectly via cognitive pre‐sleep arousal, emphasising that pre‐sleep cognition is the proximal mechanism linking distress primarily to difficulties initiating sleep. Further analysis of the role of emotional distress in sleep is important because sleep itself plays a crucial role in emotional processing (Goldstein and Walker 2014), suggesting a vicious cycle in which distress and poor sleep reinforce one another. This cycle might be mitigated by targeting pre‐sleep arousal as a modifiable mechanism.

### 4.1 Practical Implications

According to our results, interventions for students should address both chronic and daily stress processes: reducing chronically elevated stress to support more stable sleep duration, and providing strategies to manage particularly stressful days to preserve subjective sleep quality. Our results further suggest that interventions targeting dysfunctional cognitive processes, such as pre‐sleep arousal, may be particularly beneficial. There is limited evidence that cognitive‐behavioural therapy for insomnia (CBT‐I) reduces arousal‐related factors (Parsons et al. 2021), and some studies showed benefits of mindfulness‐based approaches (Hassirim et al. 2019; Ong et al. 2008). Generally, a meta‐analysis has shown that both approaches are effective in improving students' sleep, with CBT‐I yielding larger effects (Friedrich and Schlarb 2018). These interventions can also be delivered online and in brief formats. For example, Pickett et al. (2022) examined brief, online mindfulness and relaxation interventions to reduce stress and improve sleep and found promising results. Further practical implications include the development of just‐in‐time adaptive interventions (Nahum‐Shani et al. 2018) ‐ for example, app‐based prompts or brief evening routines ‐ that may help students to manage stress on particularly demanding days. Universities could also integrate low‐threshold sleep health programs into student support services, offering accessible resources such as online modules or workshops to support stress regulation and coping with pre‐sleep arousal.

### 4.2 Limitations and Strengths

Several limitations should be considered when interpreting the results of this study. Daily stress was assessed retrospectively each morning using a single item, which may have been influenced by participants' mood or prior sleep quality, particularly given the bidirectional relationship between stress and sleep (Slavish et al. 2021; Yap et al. 2020). Moreover, the temporal order of assessments limits causal interpretations. Stress, cognitive pre‐sleep arousal, and subjective sleep measures were assessed at the same time in the morning, referring to the previous day, whereas actigraphy‐based sleep duration referred to the preceding night. Thus, the timing of assessments did not align with the periods they captured. Future studies should aim to temporally separate stress and sleep assessments, ideally by measuring stress in the evening or using additional physiological indicators such as salivary cortisol (Schmidt et al. 2023), while maintaining feasibility for participants. Sensitivity analyses including additional covariates showed that the effect of daily stress on sleep parameters was not fully stable but depended on model specification, including the between‐person level of stress, weekday‐weekend differences ‐ where weekends were associated with higher subjective sleep quality, shorter SOL, and lower cognitive pre‐sleep arousal ‐ and carry‐over effects from the previous night's sleep, which were present for all outcomes except objective sleep duration. Regarding mediation analyses, the indirect effects were statistically robust, yet their implications should be considered tentative and interpreted with caution due to the only partially acceptable model fit. The suboptimal fit suggests that some pathways ‐ particularly at the between‐person level ‐ may be misrepresented, meaning that the parameter estimates provide only preliminary indications and require more comprehensive examination in future research. Generally, the sample was not representative, consisting mainly of female psychology students. Previous research has shown gender and academic‐major differences in personality traits (Vedel et al. 2015) and we also found sex differences in daily stress and all sleep outcomes in our study. While scores for perfectionistic concerns and perfectionistic strivings did not differ between women and men and were overall on a moderate level, this sample had higher neuroticism scores (*M* = 3.10) than a representative German sample (*M* = 2.25; *t*(371) = 8.76, *p* < 0.001 (Rammstedt et al. 2014). Given the strong correlation between perfectionistic concerns and neuroticism (*r* = 0.63), this might affect the generalisability of our result. External factors, particularly end‐of‐semester exam stress, which has been linked to poor sleep quality (Bouloukaki et al. 2023) may have masked potential associations between perfectionism and sleep. Participants in our study also reported heightened stress in post‐study feedback, suggesting that situational influences may have overridden the effects of more stable personality traits. As a final point, it should be mentioned that in comparison with polysomnography, the Fitbit Alta HR identifies total sleep time with good accuracy, although they have the tendency to slight overestimations (Haghayegh et al. 2019). However, measures of SOL or sleep efficiency are reported to be less accurate (e.g., Moreno‐Pino et al. 2019), which is why we did not include them in our analyses. Future research with newer sleep staging models of wearables should consider objective measures of SOL and sleep efficiency alongside subjective measures.

Despite these limitations, the study has several strengths. Generally, the results demonstrated the necessity of multilevel modelling when exploring sleep data in students, as 74%–82% of the variance in the sleep parameters was due to variability within individuals. The intensive 14‐day diary design enabled both within‐ and between‐person analyses, capturing fluctuations in stress and sleep more accurately than retrospective methods. Combining subjective reports with objective Fitbit data helped reduce common method bias, aligning with best practices in sleep‐stress research (Slavish et al. 2021). Although perfectionism could have been measured more comprehensively, the perfectionistic concerns–perfectionistic strivings distinction follows current literature standards. Finally, the naturalistic setting and brief, non‐invasive instruments enhanced ecological validity and captured sleep‐stress dynamics in students' everyday lives.

### 4.3 Conclusion

Understanding students' sleep requires a perspective that takes both individual traits and environmental stressors into account. This study suggests that perfectionistic concerns, despite its theoretical proximity to cognitive models of insomnia, did not emerge as vulnerability factor in everyday academic life. In contrast, stress emerged as important predictor of sleep, with chronic stress being more strongly related to shorter objective sleep duration and daily stress to reduced subjective sleep quality. Cognitive activity in terms of pre‐sleep arousal was observed in linking daytime stress with nighttime sleep, providing preliminary evidence that proximal cognitive processes affect students' sleep. This finding suggests that sleep‐related interventions for students should address cognitive arousal by helping them calm their minds before sleep, especially during times of high academic pressure. Approaches such as mindfulness training or cognitive strategies to manage intrusive thoughts could be especially useful. Future research should continue to explore how stress and sleep interact and how these insights can inform personalised support for better sleep in university students.

## Funding

The authors have nothing to report.

## Ethics Statement

Ethical approval was obtained by the ethics commission of the Faculty of Behavioral and Cultural Studies at Heidelberg University, Germany.

## Conflicts of Interest

The authors declare no conflicts of interest.

## Supporting information


Supporting Information S1

Supporting Information S2

Supporting Information S3

Supporting Information S4

Supporting Information S5

## Data Availability

The data that support the findings of this study are openly available in OpenScienceFramework at https://osf.io/p3yvn/, reference number 10.17605/OSF.IO/P3YVN.
