# Supplementary material for: What Role Do Perfectionism and Cognitive Pre‐Sleep Arousal Play in the Link Between Stress and Sleep? A Daily Diary Study in University Students
Source: Stress Health. 2026 Feb 5;42(1):e70136. doi: 10.1002/smi.70136 (PMC12875018; doi:10.1002/smi.70136)
Supplement: Supplementary file 1 — Supporting Information S1 [file SMI-42-e70136-s001.docx]

**Supplementary Material S1: Results of stepwise multilevel models for all sleep parameters**

| Variables | | Null model | | Random-intercept-fixed-slope-model | | Random-intercept-random slope-model | |
| --- | --- | --- | --- | --- | --- | --- | --- |
| Fixed effects | | *b* | *SE* | *b* | *SE* | *b* | *SE* |
|  | Intercept | 428.77*** | (3.34) | 451.40*** | (10.92) | 450.58*** | (10.91) |
|  | Daily stress |  |  | -0.21* | (0.10) | -0.21* | (0.10) |
|  | Perfectionistic concerns |  |  | 0.00 | (0.49) | -0.01 | (0.49) |
|  | Perfectionistic strivings |  |  | -0.25 | (0.79) | -0.18 | (0.79) |
|  | Emotional distress |  |  | -4.04* | (1.62) | -3.93* | (1.62) |
|  | Neuroticism |  |  | 4.24 | (4.95) | 3.86 | (4.94) |
|  | Extraversion |  |  | 2.44 | (3.51) | 1.92 | (3.50) |
|  | Openness |  |  | 0.94 | (3.43) | 0.70 | (3.42) |
|  | Agreeableness |  |  | -4.42 | (4.06) | -3.99 | (4.06) |
|  | Conscientiousness |  |  | -4.09 | (5.31) | -3.97 | (5.30) |
|  | Sex |  |  | -18.06* | (8.38) | -17.43* | (8.38) |
| Random variances | |  |  |  |  |  |  |
|  | Intercept σ_μ0_ | 733.10 |  | 590.10 |  | 593.20 |  |
|  | Daily stress σ_μ1_ |  |  |  |  | 0.08 |  |
|  | Residual σ_ε_ | 3342.90 |  | 3328.80 |  | 3305.00 |  |
| AIC | | 13072.40 |  | 13073.80 |  | 13077.10 |  |
| BIC | | 13087.60 |  | 13139.80 |  | 13153.30 |  |
| Conditional *R²* | |  |  | 0.18 |  | 0.19 |  |
| Marginal *R²* | |  |  | 0.04 |  | 0.04 |  |

**Table S1.1** Multilevel Models Predicting Objective Sleep Duration

*Note.* *N* = 88, 1182 days. Unstandardized estimates are displayed with standard errors given in parentheses. Objective sleep duration was measured in minutes. Sex, emotional distress, neuroticism, extraversion, openness, agreeableness and conscientiousness were entered as control variables. While sex was coded as 1 for women and 2 for men, higher values on the remaining variables indicate a stronger expression of the property. Daily stress was group-mean centered, all other predictors were grand-mean centered. **p* < .05; ***p* < .01; ****p* < .001.

**Table S1.2** Multilevel Models Predicting Subjective Sleep Quality

| Variables | Null model | | Random-intercept-fixed-slope-model | | Random-intercept-random-slope-model | | Cross-level interaction model | |
| --- | --- | --- | --- | --- | --- | --- | --- | --- |
| Fixed effects | *b* | *SE* | *b* | *SE* | *b* | *SE* | *b* | *SE* |
| Intercept | 65.01*** | (1.08) | 58.82*** | (3.56) | 58.64*** | (3.54) | 58.64*** | (3.56) |
| Daily stress |  |  | -0.09*** | (0.03) | -0.09** | (0.03) | -0.09** | (0.03) |
| Perfectionistic concerns |  |  | 0.09 | (0.16) | 0.09 | (0.16) | 0.09 | (0.16) |
| Perfectionistic strivings |  |  | 0.15 | (0.26) | 0.16 | (0.26) | 0.15 | (0.26) |
| Emotional distress |  |  | -0.26 | (0.53) | -0.23 | (0.52) | -0.23 | (0.52) |
| Neuroticism |  |  | -2.13 | (1.62) | -2.17 | (1.61) | -2.18 | (1.61) |
| Extraversion |  |  | 0.14 | (1.14) | 0.09 | (1.14) | 0.10 | (1.14) |
| Openness |  |  | -0.90 | (1.12) | -0.94 | (1.11) | -0.94 | (1.11) |
| Agreeableness |  |  | -0.04 | (1.32) | 0.03 | (1.32) | 0.03 | (1.32) |
| Conscientiousness | |  | 1.78 | (1.73) | 1.73 | (1.72) | 1.72 | (1.72) |
| Sex |  |  | 4.97 | (2.73) | 5.12 | (1.72) | 5.11 | (2.73) |
| Daily stress x Perfectionistic concerns |  |  |  |  |  |  | 0.003 | (0.00) |
| Daily stress x Perfectionistic strivings |  |  |  |  |  |  | -0.003 | (0.01) |
| Random variances |  |  |  |  |  |  |  |  |
| Intercept σμ0 | 85.53 |  | 71.74 |  | 71.62 |  | 72.27 |  |
| Daily stress σμ1 |  |  |  |  | 0.02 |  | 0.02 |  |
| Residual σε | 238.93 |  | 236.37 |  | 228.89 |  | 228.94 |  |
| AIC | 13072.40 |  | 10183.90 |  | 10180.70 |  | 10184.00 |  |
| BIC | 13087.60 |  | 10250.20 |  | 10257.10 |  | 10270.60 |  |
| Conditional R² |  |  | 0.27 |  | 0.29 |  | 0.30 |  |
| Marginal R² |  |  | 0.05 |  | 0.05 |  | 0.05 |  |

*Note.* *N* = 88, 1206 days. Unstandardized estimates are displayed with standard errors given in parentheses. Sleep quality was measured using a scale from 0 to 100 with higher values indicating a better sleep quality. Sex, emotional distress, neuroticism, extraversion, openness, agreeableness and conscientiousness were entered as control variables. While sex was coded as 1 for women and 2 for men, higher values on the remaining variables indicate a stronger expression of the property. Daily stress was group-mean centered, all other predictors were grand-mean centered. **p* < .05; ***p* < .01; ****p* < .001.

**Table S1.3** Multilevel Models Predicting Subjective Sleep Onset Latency

| Variables | | Null model | | Random-intercept-fixed-slope-model | | Random-intercept-random slope-model | |
| --- | --- | --- | --- | --- | --- | --- | --- |
| Fixed effects | | *b* | *SE* | *b* | *SE* | *b* | *SE* |
|  | Intercept | 4.07*** | (0.11) | 5.14*** | (0.36) | 5.09*** | (0.36) |
|  | Daily stress |  |  | 0.01* | (0.00) | 0.01* | (0.00) |
|  | Perfectionistic concerns |  |  | -0.02 | (0.02) | -0.02 | (0.02) |
|  | Perfectionistic strivings |  |  | 0.04 | (0.03) | 0.04 | (0.03) |
|  | Emotional distress |  |  | -0.06 | (0.05) | -0.06 | (0.05) |
|  | Neuroticism |  |  | 0.21 | (0.16) | 0.21 | (0.16) |
|  | Extraversion |  |  | 0.02 | (0.12) | 0.03 | (0.11) |
|  | Openness |  |  | 0.08 | (0.11) | 0.07 | (0.11) |
|  | Agreeableness |  |  | -0.01 | (0.13) | -0.04 | (0.13) |
|  | Conscientiousness |  |  | -0.15 | (0.17) | -0.17 | (0.17) |
|  | Sex |  |  | -0.86** | (0.28) | -0.82** | (0.27) |
| Random variances | |  |  |  |  |  |  |
|  | Intercept σ_μ0_ | 0.96 |  | 0.75 |  | 0.74 |  |
|  | Daily stress σ_μ1_ |  |  |  |  | 0.00 |  |
|  | Residual σ_ε_ | 2.22 |  | 2.21 |  | 2.21 |  |
| AIC | | 4543.30 |  | 4541.30 |  | 4544.10 |  |
| BIC | | 4558.60 |  | 4607.50 |  | 4620.50 |  |
| Conditional *R²* | |  |  | 0.30 |  | NA |  |
| Marginal *R²* | |  |  | 0.07 |  | 0.08 |  |

*Note.* *N* = 88, 1202 days. Unstandardized estimates are displayed with standard errors given in parentheses. Subjective sleep onset latency (SOL) was measured in minutes and transformed by square root transformation. Sex, emotional distress, neuroticism, extraversion, openness, agreeableness and conscientiousness were entered as control variables. While sex was coded as 1 for women and 2 for men, higher values on the remaining variables indicate a stronger expression of the property. Daily stress was group-mean centered, all other predictors were grand-mean centered. Due to singularity issues, the conditional *R²* was not available (NA) for the random-intercept-random-slope model. **p* < .05; ***p* < .01; ****p* < .001.

**Table S1.4** Multilevel Models Predicting Cognitive Pre-Sleep Arousal

|  | | Null model | | Random-intercept-fixed-slope-model | | Random-intercept-random slope-model | |
| --- | --- | --- | --- | --- | --- | --- | --- |
| Fixed effects | | *b* | *SE* | *b* | *SE* | *b* | *SE* |
|  | Intercept | 12.00*** | (0.39) | 12.37*** | (1.04) | 12.52*** | (0.99) |
|  | Daily stress |  |  | 0.06*** | (0.01) | 0.06*** | (0.01) |
|  | Perfectionistic concerns |  |  | 0.06 | (0.05) | 0.06 | (0.04) |
|  | Perfectionistic strivings |  |  | 0.02 | (0.07) | 0.02 | (0.07) |
|  | Emotional distress |  |  | 0.49** | (0.15) | 0.51*** | (0.14) |
|  | Neuroticism |  |  | 0.91 | (0.47) | 0.59 | (0.44) |
|  | Extraversion |  |  | -0.37 | (0.33) | -0.29 | (0.31) |
|  | Openness |  |  | -0.30 | (0.33) | -0.38 | (0.31) |
|  | Agreeableness |  |  | 0.67 | (0.39) | 0.59 | (0.36) |
|  | Conscientiousness |  |  | 0.00 | (0.50) | -0.12 | (0.48) |
|  | Sex |  |  | -0.31 | (0.80) | -0.43 | (0.75) |
| Random variances | |  |  |  |  |  |  |
|  | Intercept σ_μ0_ | 12.22 |  | 6.34 |  | 6.49 |  |
|  | Daily stress σ_μ1_ |  |  |  |  | 0.00 |  |
|  | Residual σ_ε_ | 18.14 |  | 17.00 |  | 16.02 |  |
| AIC | | 7128.00 |  | 7024.60 |  | 7001.50 |  |
| BIC | | 7143.30 |  | 7090.80 |  | 7077.90 |  |
| Conditional *R²* | |  |  | 0.44 |  | 0.46 |  |
| Marginal *R²* | |  |  | 0.23 |  | 0.20 |  |

*Note.* *N* = 88, 1206 days. Unstandardized estimates are displayed with standard errors given in parentheses. Sex, emotional distress, neuroticism, extraversion, openness, agreeableness and conscientiousness were entered as control variables. While sex was coded as 1 for women and 2 for men, higher values on the remaining variables indicate a stronger expression of the property. Daily stress was group-mean centered, all other predictors were grand-mean centered. Due to singularity issues, the conditional *R²* was not available (NA) for the random-intercept-random-slope model. **p* < .05; ***p* < .01; ****p* < .001.
