# Supplementary material for: What Role Do Perfectionism and Cognitive Pre‐Sleep Arousal Play in the Link Between Stress and Sleep? A Daily Diary Study in University Students
Source: Stress Health. 2026 Feb 5;42(1):e70136. doi: 10.1002/smi.70136 (PMC12875018; doi:10.1002/smi.70136)
Supplement: Supplementary file 2 — Supporting Information S2 [file SMI-42-e70136-s002.docx]

**Supplementary Material S2: Sensitivity Analyses**

| Variables | Objective sleep duration | | Subjective sleep quality | | Subjective SOL | | Cognitive pre-sleep arousal | |
| --- | --- | --- | --- | --- | --- | --- | --- | --- |
| Fixed effects | *b* | *SE* | *b* | *SE* | *b* | *SE* | *b* | *SE* |
| Intercept | 454.30*** | (11.55) | 59.01*** | (3.50) | 5.26*** | (0.37) | 12.77*** | (1.09) |
| Daily stress | -0.19 | (0.10) | -0.07** | (0.03) | 0.01* | (0.00) | 0.06*** | (0.01) |
| Perfectionistic concerns | -0.02 | (0.52) | 0.06 | (0.16) | -0.02 | (0.02) | 0.06 | (0.05) |
| Perfectionistic strivings | 0.00 | (0.84) | 0.26 | (0.25) | 0.04 | (0.03) | 0.04 | (0.08) |
| Emotional distress | -4.77** | (1.71) | -0.29 | (0.52) | -0.07 | (0.05) | 0.47** | (0.16) |
| Neuroticism | 3.91 | (5.23) | -2.40 | (1.59) | 0.24 | (0.17) | 0.91 | (0.49) |
| Extraversion | 3.31 | (3.72) | 0.01 | (1.13) | 0.05 | (0.12) | -0.31 | (0.35) |
| Openness | 0.03 | (3.63) | -1.16 | (1.10) | 0.06 | (0.12) | -0.40 | (0.34) |
| Agreeableness | -3.69 | (4.31) | -0.17 | (1.30) | -0.01 | (0.14) | 0.63 | (0.41) |
| Conscientiousness | -4.17 | (5.68) | 1.41 | (1.71) | -0.17 | (0.18) | -0.06 | (0.53) |
| Sex | -17.96* | (8.87) | 4.93 | (2.69) | -0.91** | (0.28) | -0.50 | (0.84) |
| Random variances |  |  |  |  |  |  |  |  |
| Intercept σμ0 | 682.90 |  | 67.00 |  | 0.77 |  | 6.90 |  |
| Residual σε | 3029.50 |  | 231.50 |  | 2.22 |  | 17.46 |  |
| Conditional *R²* | 0.22 |  | 0.27 |  | 0.32 |  | 0.45 |  |
| Marginal *R²* | 0.05 |  | 0.06 |  | 0.08 |  | 0.23 |  |

**Table S2.1** Results of the Final Models Predicting Daily Sleep Outcomes Excluding Days of Alcohol or Cannabis Consumption

*Note.* *N* = 88, 1054 days for objective sleep duration (random-intercept fixed-slope model), 1076 days for subjective sleep quality (random-intercept fixed-slope model), 1072 days for subjective sleep onset latency (SOL; random-intercept fixed-slope model) and 1076 days for cognitive pre-sleep arousal (random-intercept fixed-slope model). Unstandardized estimates are displayed with standard errors given in parentheses. Objective sleep duration and subjective SOL were measured in minutes and SOL was root-transformed. Sleep quality was assessed using a scale from 0 to 100 with higher values indicating a better sleep quality. Sex, emotional distress, neuroticism, extraversion, openness, agreeableness and conscientiousness were entered as control variables. While sex was coded as 1 for women and 2 for men, higher values on the remaining variables indicate a stronger expression of the property. Daily stress was group-mean centered, all other predictors were grand-mean centered.

**p* < .05; ***p* < .01; ****p* < .001.

**Table S2.2** Results of the Final Models Predicting Daily Sleep Outcomes Excluding Participants who Indicated a Critical Life Event that Drastically Affected their Sleep Behavior

| Variables | Objective sleep duration | | Subjective sleep quality | | Subjective SOL | | Cognitive pre-sleep arousal | |
| --- | --- | --- | --- | --- | --- | --- | --- | --- |
| Fixed effects | *b* | *SE* | *b* | *SE* | *b* | *SE* | *b* | *SE* |
| Intercept | 427.99*** | (3.45) | 59.90*** | (3.53) | 5.16*** | (0.37) | 12.44*** | (1.06) |
| Daily stress |  |  | -0.08* | (0.03) | 0.01* | (0.00) | 0.06*** | (0.01) |
| Perfectionistic concerns |  |  | 0.15 | (0.16) | -0.03 | (0.02) | 0.04 | (0.05) |
| Perfectionistic strivings |  |  | 0.06 | (0.26) | 0.05 | (0.03) | 0.06 | (0.08) |
| Emotional distress |  |  | -0.24 | (0.57) | -0.03 | (0.06) | 0.58** | (0.17) |
| Neuroticism |  |  | -3.11 | (1.61) | 0.25 | (0.17) | 0.99* | (0.48) |
| Extraversion |  |  | 0.61 | (1.12) | 0.00 | (0.12) | -0.46 | (0.34) |
| Openness |  |  | -0.50 | (1.10) | 0.07 | (0.11) | -0.32 | (0.33) |
| Agreeableness |  |  | 0.42 | (1.32) | -0.05 | (0.14) | 0.63 | (0.40) |
| Conscientiousness |  |  | 2.15 | (1.68) | -0.19 | (0.17) | -0.11 | (0.50) |
| Sex |  |  | 4.45 | (2.68) | -0.88** | (0.28) | -0.36 | (0.81) |
| Random variances |  |  |  |  |  |  |  |  |
| Intercept σμ0 | 768.10 |  | 66.99 |  | 0.74 |  | 6.29 |  |
| Daily stress σ_μ1_ |  |  | 0.02 |  |  |  |  |  |
| Residual σε | 3125.60 |  | 225.56 |  | 2.20 |  | 16.26 |  |
| Conditional *R²* | 0.20 |  | 0.29 |  | 0.31 |  | 0.46 |  |
| Marginal *R²* | 0.00 |  | 0.06 |  | 0.07 |  | 0.26 |  |

*Note.* *N* = 84, 1133 days for objective sleep duration (intercept-only model), 1154 days for subjective sleep quality (random-intercept random-slope model), 1150 days for subjective sleep onset latency (SOL; random-intercept fixed-slope model), and 1154 days for cognitive pre-sleep arousal (random-intercept fixed-slope model). Unstandardized estimates are displayed with standard errors given in parentheses. Objective sleep duration and subjective SOL were measured in minutes and SOL was root-transformed. Sleep quality was assessed using a scale from 0 to 100 with higher values indicating a better sleep quality. Sex, emotional distress, neuroticism, extraversion, openness, agreeableness and conscientiousness were entered as control variables. While sex was coded as 1 for women and 2 for men, higher values on the remaining variables indicate a stronger expression of the property. Daily stress was group-mean centered, all other predictors were grand-mean centered.

**p* < .05; ***p* < .01; ****p* < .001.

**Table S2.3** Results of the Final Models Predicting Daily Sleep Outcomes Including Additional Covariates

| Variables | Objective sleep duration | | Subjective sleep quality | | Subjective SOL | | Cognitive pre-sleep arousal | |
| --- | --- | --- | --- | --- | --- | --- | --- | --- |
| Fixed effects | *b* | *SE* | *b* | *SE* | *b* | *SE* | *b* | *SE* |
| Intercept | 479.50*** | (18.25) | 55.06*** | (3.97) | 4.58*** | (0.37) | 9.76*** | (0.94) |
| Lag-1 outcome | -0.05 | (0.03) | 0.07* | (0.03) | 0.09** | (0.03) | 0.09** | (0.03) |
| Daily stress | -0.18 | (0.10) | -0.08** | (0.03) | 0.00 | (0.00) | 0.05*** | (0.01) |
| Average stress | -0.48* | (0.23) | -0.07 | (0.07) | 0.01 | (0.01) | 0.10*** | (0.02) |
| Perfectionistic concerns | 0.00 | (0.51) | 0.06 | (0.15) | -0.02 | (0.01) | 0.06 | (0.04) |
| Perfectionistic strivings | -0.10 | (0.82) | 0.18 | (0.25) | 0.03 | (0.02) | -0.08 | (0.06) |
| Emotional distress | -3.16 | (1.69) | -0.12 | (0.52) | -0.06 | (0.05) | 0.24 | (0.13) |
| Neuroticism | 5.50 | (5.05) | -1.60 | (1.55) | 0.15 | (0.15) | 0.65 | (0.39) |
| Extraversion | 0.91 | (3.63) | -0.38 | (1.11) | 0.06 | (0.10) | -0.09 | (0.28) |
| Openness | 1.88 | (3.54) | -0.73 | (1.08) | 0.03 | (0.10) | -0.56* | (0.27) |
| Agreeableness | -7.88 | (4.21) | -0.09 | (1.28) | 0.05 | (0.12) | 0.94** | (0.32) |
| Conscientiousness | -0.95 | (5.50) | 2.37 | (1.68) | -0.21 | (0.16) | -0.36 | (0.42) |
| Sex | -24.47** | (8.98) | 3.58 | (2.73) | -0.65* | (0.26) | 1.07 | (0.69) |
| Weekend | 6.15 | (3.81) | 4.18*** | (1.01) | -0.38*** | (0.10) | -0.90*** | (0.26) |
| Random variances |  |  |  |  |  |  |  |  |
| Intercept σμ0 | 593.40 |  | 61.96 |  | 0.55 |  | 3.82 |  |
| Residual σε | 3233.90 |  | 234.22 |  | 2.20 |  | 15.70 |  |
| Conditional *R²* | 0.22 |  | 0.26 |  | 0.28 |  | 0.46 |  |
| Marginal *R²* | 0.05 |  | 0.07 |  | 0.10 |  | 0.33 |  |

*Note.* *N* = 88; All models were random-intercept fixed-slope models; 1061 days for objective sleep duration, 1100 days for subjective sleep quality, 1092 days for subjective sleep onset latency, and 1100 days for cognitive pre-sleep arousal. Unstandardized estimates are displayed with standard errors given in parentheses. Objective sleep duration and subjective SOL were measured in minutes and SOL was root-transformed. Sleep quality was assessed using a scale from 0 to 100 with higher values indicating a better sleep quality. Sex, weekend, the respective previous night outcome variable (lag-1 outcome), average stress (i.e., participant’s mean value across all assessed days), emotional distress, neuroticism, extraversion, openness, agreeableness, and conscientiousness were entered as control variables. While sex was coded as 1 for women and 2 for men and weekend as 1 for weekend days and 0 for weekdays, higher values on the remaining variables indicate a stronger expression of the property. Daily stress was group-mean centered (within-person); all other predictors were grand-mean centered (between-person).

**p* < .05; ***p* < .01; ****p* < .001.
