# Supplementary figures and images for: What Role Do Perfectionism and Cognitive Pre‐Sleep Arousal Play in the Link Between Stress and Sleep? A Daily Diary Study in University Students
Source: Stress Health. 2026 Feb 5;42(1):e70136. doi: 10.1002/smi.70136 (PMC12875018; doi:10.1002/smi.70136)

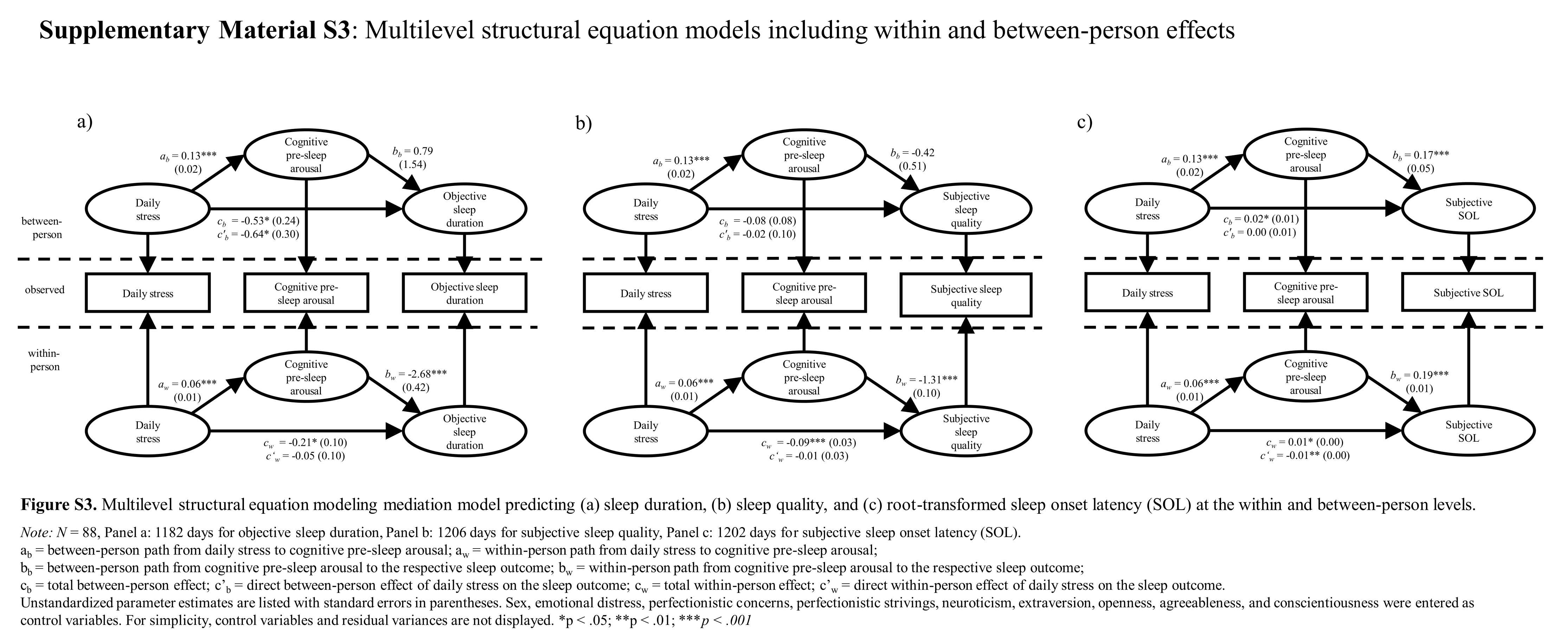

Supplement: Supplementary file 3 — Supporting Information S3 [file SMI-42-e70136-s004.png]
