# Supplementary material for: What Role Do Perfectionism and Cognitive Pre‐Sleep Arousal Play in the Link Between Stress and Sleep? A Daily Diary Study in University Students
Source: Stress Health. 2026 Feb 5;42(1):e70136. doi: 10.1002/smi.70136 (PMC12875018; doi:10.1002/smi.70136)
Supplement: Supplementary file 4 — Supporting Information S4 [file SMI-42-e70136-s003.docx]

**Supplementary Material S4:** Model fit indices for the three mediation models predicting different sleep outcomes

**Table S4.** Model fit indices

| **Sleep Outcome** | **χ² (df)** | ***p*-value** | **CFI** | **TLI** | **RMSEA** | **SRMR_within_** | **SRMR_between_** |
| --- | --- | --- | --- | --- | --- | --- | --- |
| Objective sleep duration | 81.19 (18) | < .001 | 0.817 | 0.665 | 0.054 | 0.000 | 0.159 |
| Subjective sleep quality | 83.81 (18) | < .001 | 0.821 | 0.672 | 0.055 | 0.000 | 0.160 |
| Subjective sleep latency | 83.23 (18) | < .001 | 0.887 | 0.793 | 0.055 | 0.000 | 0.169 |

*Note:* Model fit was considered acceptable if the chi-square test was not significant (*p* > .05), the comparative fit index (CFI) and Tucker–Lewis index (TLI) were ≥ .95, the root mean square error of approximation (RMSEA) was < .06, and the standardized root mean square residual (SRMR) was < .08.
