# Supplementary material for: What Role Do Perfectionism and Cognitive Pre‐Sleep Arousal Play in the Link Between Stress and Sleep? A Daily Diary Study in University Students
Source: Stress Health. 2026 Feb 5;42(1):e70136. doi: 10.1002/smi.70136 (PMC12875018; doi:10.1002/smi.70136)
Supplement: Supplementary file 5 — Supporting Information S5 [file SMI-42-e70136-s005.docx]

**Supplementary Material S5: Explorative Analysis on Emotional Distress**

**
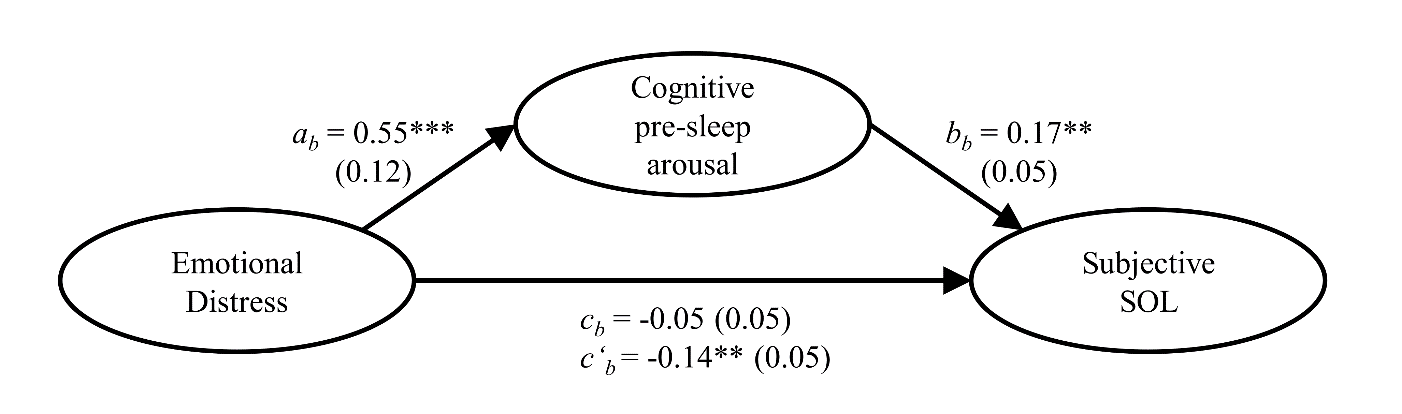
**

**Figure S5.** Multilevel Structural Equation Modeling Mediation Model Predicting Subjective Sleep Onset Latency.

*Note:* Subjective sleep onset latency (SOL) was square-root transformed. Unstandardized parameter estimates are listed with standard errors in parentheses. The figure displays between-person effects only; within-person effects of daily stress on both the mediator and the outcome were included in the model but are not shown here. Sex, perfectionistic concerns, perfectionistic strivings, neuroticism, extraversion, openness, agreeableness, and conscientiousness were entered as control variables. For simplicity, control variables and residual variances are not displayed. The path c refers to the total effect and c′ to the direct effect. **p* < .05; ***p* < .01; ****p* < .001.

**Table S5.** Model fit indices

| **Chi Square** | χ²(33) = 611.50, *p* < .001 |
| --- | --- |
| **CFI** | 0.917 |
| **TLI** | 0.839 |
| **RMSEA** | 0.048 |
| **SRMR_within_** | 0.000 |
| **SRMR_between_** | 0.129 |

*Note:* Model fit was considered acceptable if the chi-square test was not significant (*p* > .05), the comparative fit index (CFI) and Tucker–Lewis index (TLI) were ≥ .95, the root mean square error of approximation (RMSEA) was < .06, and the standardized root mean square residual (SRMR) was < .08.
